# Examination of Thoracic and Lumbosacral Spine Guide for Neurosurgery Residents

**DOI:** 10.21315/mjms2023.30.2.10

**Published:** 2023-04-18

**Authors:** Mohd Arman Muhamad Nor, Mohammad Imran Ahmad, Anis Nabillah Mohd Azli, Jafri Malin Abdullah, Zamzuri Idris, Abdul Rahman Izaini Ghani

**Affiliations:** 1 Department of Neurosciences, School of Medical Sciences, Universiti Sains Malaysia, Kelantan, Malaysia; 2 Brain and Behaviour Cluster, School of Medical Sciences, Universiti Sains Malaysia, Kelantan, Malaysia; 3 Department of Neurosurgery, Institut Kajisaraf Tunku Abdul Rahman (IKTAR), Hospital Kuala Lumpur, Kuala Lumpur, Malaysia; 4 Neurosurgery Unit, Hospital Sultanah Nur Zahirah, Terengganu, Malaysia

**Keywords:** examination technique, thoracic, lumbosacral spine, range of motion, rotation

## Abstract

**Background:**

This paper outlines a summary of examination techniques for the thoracic and lumbosacral spine. It starts with observation, palpation and a range of movements followed by various special tests to identify thoracic and lumbosacral spine pathology.

**Methods:**

Bedside instruments used include a measuring tape, scoliometer and back range of motion instrument (BROM II).

**Discussion:**

Back flexion-extension, lateral flexion and rotation were assessed with bedside instruments. This would aid in increasing the accuracy and precision of objective measurement while conducting a clinical examination to determine the back range motion. Specific tests were used to localise specific anatomical locations and identify the spine pathology that can help the clinician to diagnose and treat the disease.

## Introduction

### Anatomy of Thoracic and Lumbosacral Spine

The spinal vertebrae function both as the central mechanical support for the body and as protection for the spinal cord. The vertebral bodies are separated by a connective tissue layer called intervertebral discs, which consist of a central nucleus pulposus surrounded by an outer capsule called annulus fibrosus. Posteriorly, the neural elements are surrounded by an arch of bone formed by the pedicles, transverse processes, laminae, and spinous processes. The superior and inferior articular processes or facet joints form additional points of mechanical contact between the adjacent vertebrae. The spinal cord runs through the spinal canal (vertebral foramen) and is surrounded by pia, arachnoid and dura mater. The nerve roots exit the spinal canal via the neural (intervertebral) foramina (1).

### General Inspection

#### Observation

Look at the gait once the patient walks into the clinic. Note for any deformity, abnormality of posture or motility. Then look for scars, café au lait spots, hairy patches or fat pads, as well as any lumps and bumps. Observe for the normal contour of the spine as patients with tuberculosis and neoplasms of the spine can have marked kyphosis or muscular dystrophy that often results in an increased lumbar lordosis. Scoliosis is common in syringomyelia and Friedreich’s ataxia (2). Look at the sitting posture and how the patient rises from a seated position.

#### Palpation

There may be areas of localised tenderness or muscle spasms that could be elicited on palpation. Manipulation or percussion over the spinous processes and pressure just lateral to them may reproduce and exacerbate the pain. Pain reproducible upon palpation of the spinous process may indicate a problem with the vertebrae, for example, recent fractures. Pain palpated on the paraspinal region may indicate muscle spasms, especially if tensed back muscles are present (2).

#### The Adam Test

**Figure 1:**
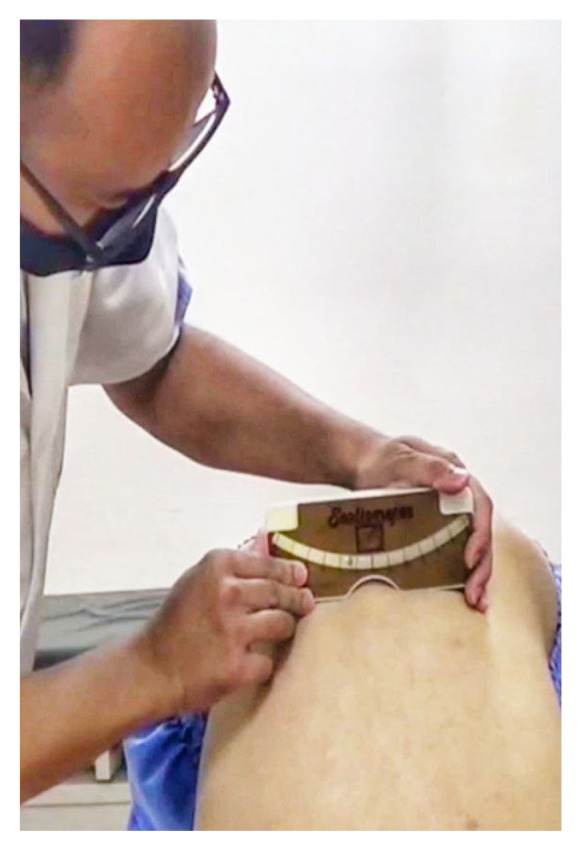
The Adam test

The first step in scoliosis examination is a simple inspection. This includes inspection of a standing patient from behind and optical evaluation of asymmetries in the shoulders, scapulae, waistline, the distance of the arms from the trunk, as well as the ‘balance’ of the head. The principal screening test for scoliosis is the physical examination of the back, which includes the Adams forward-bending test while the ‘scoliometre’ helps quantify the trunk deformation (Figure 1). In the standing forward bending position, the examined person is asked to bend forward looking down while keeping the feet approximately 15 cm apart. The scoliometre is then used in three areas of interest namely upper thoracic (T3–T4), main thoracic (T5–T12) and the thoracolumbar area (T12–L1 or L2–L3). Scoliometre measurement equal to 0° is defined as symmetry at that particular level of the trunk (3). Any other scoliometre values are defined as asymmetry.

### Range of Movements

#### Functional Test for a Range of Movements

Further provocative tests and manoeuvres may be carried out on the spine to determine normal or abnormal spinal movement and function. This part of the examination aims to look for the normal range of motion of the vertebrae as well as to identify any restrictions in the movement.

#### Equipment

Back Range of Motion Instruments (BROM II) are sets of equipment that can help accurately measure the range of motion over the back. This equipment can be fixated directly to the back of the patient, thus eliminating examiner bias and the need for a definition of a fixated point on the body (4).

### Active Range of Movement Thoraco-Lumbar

#### Thoracic Extension

**Figure 2:**
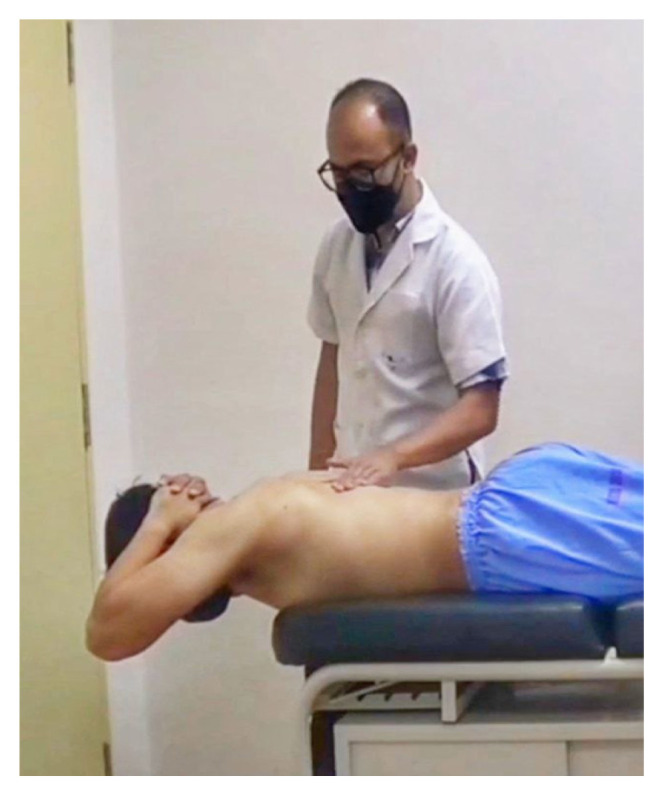
Thoracic extension

The patient is in a prone position with head and upper trunk extending off the table from about the nipple line while the examiner stabilises the lower limbs at the ankle. The patient then extends the thoracic spine to the horizontal (Figure 2). Instructions to the patient: “Raise your head, shoulders and chest to the table level” (5).

#### Lumbar Extension

**Figure 3:**
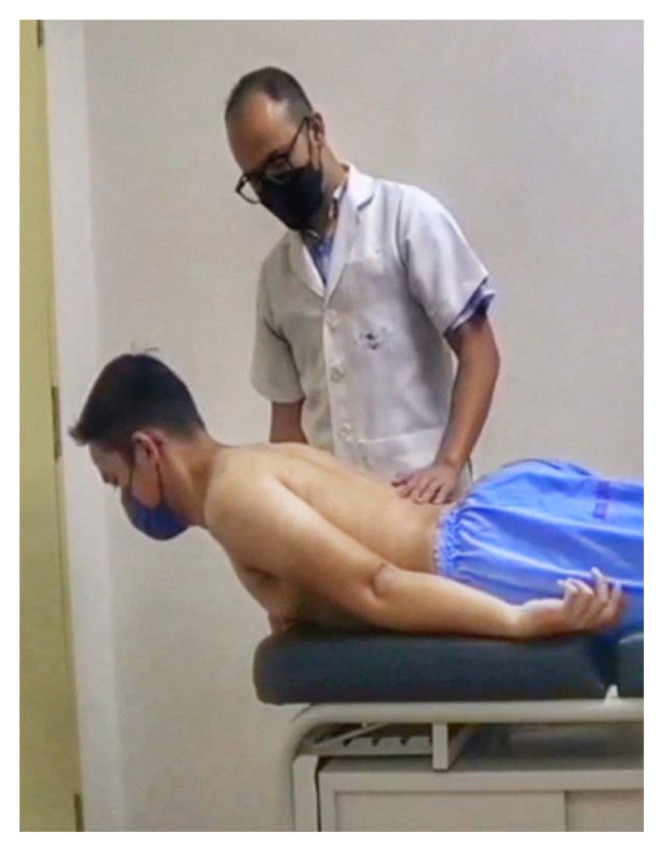
Lumbar extension

The patient is in a prone position with hands at side clasped behind the head. Examiner stabilises the lower extremities just above the ankles if the patient has normal hip strength. The patient then extends the lumbar spine until the entire thorax is raised from the table (clears umbilicus) (Figure 3). Instructions to the patient: “Raise your head, shoulders, and chest off the table. Come up as high as you can” (5).

#### Trunk Flexion

Trunk flexion has multiple elements that include both thoracic and lumbar motion. The patient is in a supine position with hands clasped behind the head. Examiner stands at the side of the table at the level of the patient’s chest to be able to ascertain whether the scapulae clear the table during the test. Instructions to the patient: “Tuck your chin and bring your head, shoulders, and arms off the table as in a sit-up” (5).

#### Trunk Rotation

The patient is in a supine position with hands clasped behind the head. Examiner stands at the patient’s waist level. The patient flexes the trunk and rotates to one side. This movement is then repeated on the opposite side so that muscles on both sides can be examined. Instructions to the patient: “Lift your head and shoulders from the table, taking your right elbow toward your left knee.” Then, “Lift your head and shoulders from the table, taking your left elbow towards your right knee” (5).

### Passive Range of Movement Thoraco-Lumbar

#### Flexion

The examiner palpates between the spinous processes using three fingers: one above, one on and one below the spinous process. The examiner then uses their body weight to perform a flexing movement in the lumbar spine and feels for the ‘gapping’ during the movement, noting if the movement is normal, hypo or hypermobile (6).

#### Extension

The patient lies in a prone position with arms crossed and head resting on the hands. The examiner uses the crossed arms to pull the patient’s trunk upwards while feeling the movements of the spinous processes and noting the normal, hypo or hypermobility.

#### Trunk Rotation

The patient is in a supine position with hands clasped behind the head. Examiner stands at the patient’s waist level. The examiner uses the crossed arms to pull the patient’s trunk upwards and rotates to one side while feeling the movements of the spinous processes and noting the normal, hypo or hypermobility (7).

This movement is then repeated on the opposite side so that the muscles on both sides can be examined.

### Grading for Thoracic and Lumbar Extension

The Grade 5 and Grade 4 tests for spine extension are different for the lumbar and thoracic spines. Beginning at Grade 3, the tests for both levels are combined (5).

Grade 0 (Zero): No contractile activity.

Grade 1 (Trace): Contractile activity is detectable but no movement.

Grade 2 (Poor): The patient completes the partial range of motion.

Grade 3 (Fair): The patient completes the range of motion.

#### Grading for Thoracic Extension (5)

Grade 4 (Good): The patient can raise the trunk to the horizontal level but does it somewhat laboriously.

Grade 5 (Normal): The patient can raise the upper trunk quickly from its forward flexed position to the horizontal (or beyond) with ease and with no sign of exertion.

#### Grading for Lumbar Extension (5)

Grade 4 (Good): The patient can come to an end position but may waver or display some signs of effort.

Grade 5 (Normal): The patient can quickly come to an end position and hold that position without evidence of significant effort.

### Grading for Trunk Flexion

Grade 5 (Normal): The patient completes the range of motion until inferior angles of the scapulae are off the table (the weight of the arms serves as a resistance).

Grade 4 (Good): Patient completes the range of motion and raises their trunk until the scapulae are off the table. Resistance of the arms is reduced in the cross-chest position.

Grade 3 (Fair): The patient completes the range of motion and flexes their trunk until the inferior angles of the scapulae are off the table.

#### Sequence for Grading Trunk Flexion Grades 0–2

Testing trunk flexion is rather a clear cut for Grades 5, 4 and 3. When testing Grade 2 and below, the results may be ambiguous but observation and palpation are critical (5).

- Head raise: Ask the patient to lift the head from the table. If the scapulae do not clear the table, the Grade is 2.
- Assisted forward lean: The examiner cradles the upper trunk and head off the table and asks the patient to lean forward. If there is a depression in the rib cage, the Grade is 2 (Poor). If there is no depression on the rib cage but visible or palpable contraction occurs, the Grade assigned should be 1 (Trace). If there is no activity, the Grade is 0.
- Ask the patient to cough. If the patient can cough to any degree and depression in the rib cage occurs, the Grade is 2 (Poor). If the patient is unable to cough but there is palpable rectus abdominis activity, the Grade is 1 (Trace). The lack of any demonstrable activity is Grade 0.

### Grading for Trunk Rotation

Grade 5 (Normal): Patient is in a supine position with hands clasped behind the head. The scapula which corresponds to the side of the external oblique function must clear the table for a normal grade.

Grade 4 (Good): Patient is in a supine position with arms crossed over the chest. Other than the patient’s position, all other aspects of the test are the same as for Grade 5.

For Grades 2 and 3: The position of the patient is supine with arms outstretched above the plane of the body (5).

Grade 3 (Fair): The patient can raise the scapula off the table.

Grade 2 (Poor): The patient is unable to clear the inferior angle of the scapula from the table on the side of the external oblique being tested. The examiner must be able to observe the depression in the rib cage.

For Grade 1 (Trace) and Grade 0 (Zero). The patient’s position is supine with arms at the sides. Hips flexed with feet flat on the table. The head is supported as the patient attempts to turn to one side (5).

Grade 1 (Trace): The examiner can see or palpate muscular contraction.

Grade 0 (Zero): No response from the obliquus internus or externus muscles.

### Special Test for Range of Movement

#### Modified Schober’s Test

**Figure 4:**
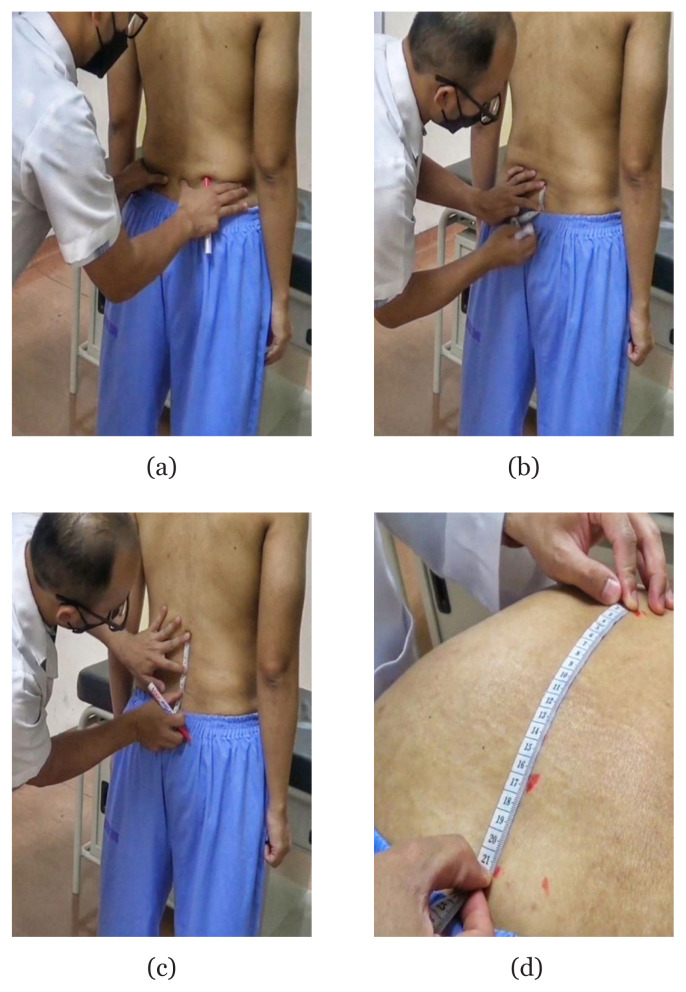
Schober’s test (a) Identify the posterior superior iliac spine (PSIS) at both sides and draw a horizontal line at the centre of these two points, (b) A second line is marked 5 cm below the horizontal line, (c) third line is marked 10 cm above the horizontal line, (d) Examiner remeasures the distance between the top and bottom line while the patient flexes forward as if attempting to touch their toes. The gap between these two marks should increase by > 5 cm

Classically used to determine if there is a decrease in lumbar spine range of motion. The patient is standing and the examiner marks both posterior superior iliac spine (PSIS) and then draws a horizontal line at the centre of both marks. A second line is marked 5 cm below the first line. A third line is marked 10 cm above the first line. The patient is then instructed to flex forward as if attempting to touch their toes while the examiner remeasures the distance between the top and the bottom line. This gap between the two marks (which is currently 15 cm) should increase by > 5 cm. Anything less than this indicates reduced lumbar flexion (9). The technique to perform Schober’s test is shown in Figure 4.

### Thoraco-Lumbosacral Spine Examination as per Anatomical Order

#### Skin

##### Surface anatomy

During palpation, the examiner should proceed with the identification of the vertebral surface anatomy. The 4 most common points are T1, T7/8, L4, and S2 landmarks. The T1 spinous process has been identified as the most prominent spinous process at the base of the neck. The T7/8 spinous process is at the level of the lower border of the scapula. The L4 spinous process is at the level of the iliac crest. The S2 vertebra is at the dimple in the midline of the back at the level of the posterior superior iliac spine. It is a rough estimation and may vary by 1 level of the vertebra (10).

#### Muscle

##### Back muscles

Testing for the weakness of the large back muscles (Table 1). The average patient’s back is far too strong for the examiner to test by manual opposition. With the patient in a prone position, ask the patient to arch the back and rock on the stomach. Inspect and palpate the paraspinal muscles.

#### Hip Muscles-Hip Abductor

##### Trendelenburg’s sign

The patient is asked to stand on one leg for 30 s without leaning to one side (Table 2). The patient can hold onto something if balance is an issue. The examiner observes the patient to see if the pelvis stays level during the single-legstance. A positive Trendelenburg test is indicated if, during unilateral weight-bearing, the pelvis drops toward the unsupported side (11).

#### Ligaments

##### Yeoman’s test

**Figure 5:**
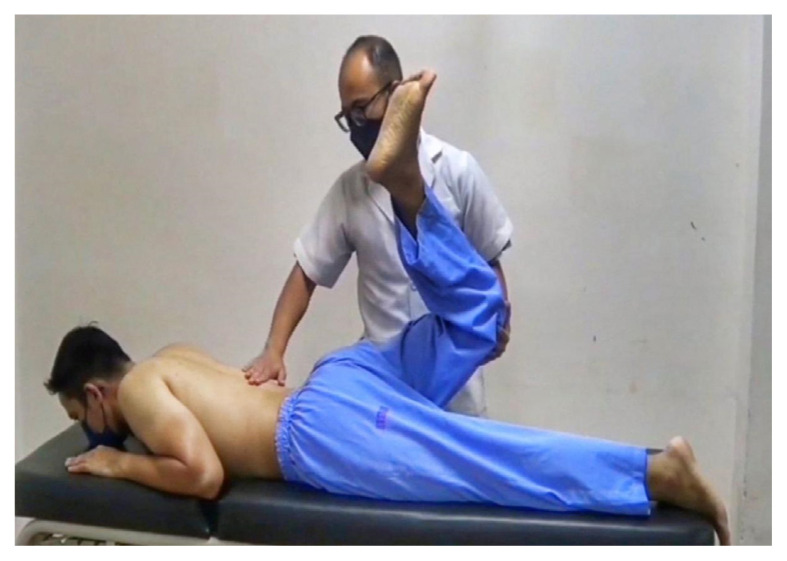
Yeoman’s test

The patient lies prone while the examiner stands at the painful side and flexes the patient’s knee to 90° and extends the hip. Pain localised to the sacroiliac joint indicates pathology in the anterior sacroiliac ligament (Figure 5). Anterior thigh paraesthesia may indicate a femoral nerve stretch (12).

##### Sacroiliac compression test

**Figure 6:**
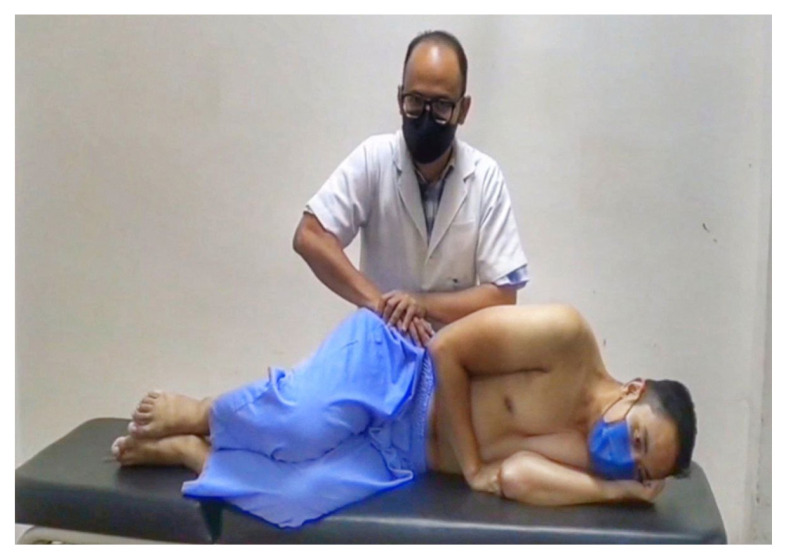
Sacroiliac compression test

This test is a pain provocation test, which stresses the sacroiliac joint (SIJ) structures, in particular, the posterior SIJ ligament, in an attempt to replicate the patient’s symptom. The patient is on their side-lying and the examiner’s hands are placed over the upper part of the iliac crest pressing toward the floor. The movement causes forward pressure on the sacrum. An increased feeling of pressure in the SIJ indicates a possible sacroiliac lesion or a sprain over the posterior sacroiliac ligaments (Figure 6). A positive result is indicated by pain or replication of the patient’s symptoms (13).

#### Facet

##### Kemp test

**Figure 7:**
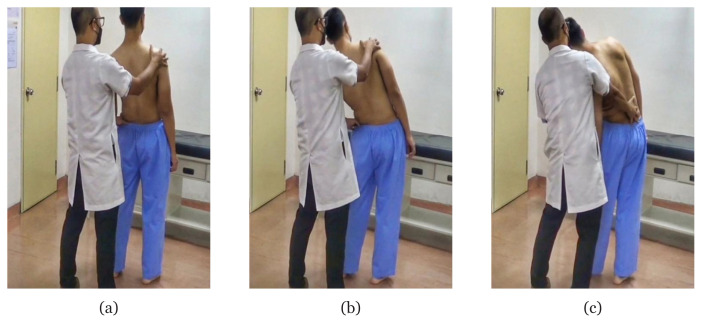
Kemp test (a) Examiner is positioning the patient to extension, (b) ipsilateral side bending and (c) ipsilateral rotation position

The patient is standing and the examiner fixes the opposite ilium from the side. While the patient is being tested with one hand, the other hand grabs the shoulder and leads the patient to extension, ipsilateral side bending and ipsilateral rotation position. Hold this position for three seconds (Figure 7). The test is positive when the patient reports pain, numbness or tingling in the back area or lower extremities. The pain is located on the side being tested. Local pain suggests a facet cause while radiating pain into the leg is more suggestive of nerve root irritation, especially if the pain is below the knee (14).

#### Bone-Interspinous Space

##### Interspinous gap change test

The patient stands with feet shoulder-width apart in front of the examination table. Then ask the patient to flex their back with both hands on the edge of the table. At flexion, inspect gaps between interspinous processes in a craniocaudal direction. An interspinous space that is bent or wider than the adjacent interspinous spaces may indicate an unstable level. Then ask the patient to extend his back and push his buttock towards the examination table with both hands on the table to reproduce lumbar extension from the flexion position. Evaluate the change in the gap of the interspinous space using both thumbs with one thumb at the suspected interspinous space that is unstable and the other thumb on one level above or below to compare the changes in the gap of the two spaces (15). The test is positive if the width of an interspinous space abruptly becomes narrow compared to other interspinous processes or if changing position of the upper spinous process is anteriorly or posteriorly from the original state (16). Tenderness is usually detected during palpation of the interspinous process that is widened.

#### Disc

##### The reverse straight leg raising (femoral stretch or Ely test)

**Figure 8:**
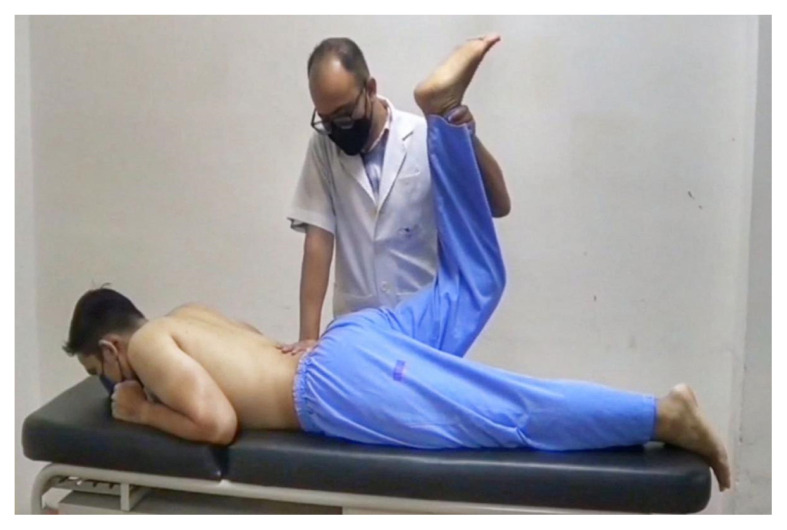
Reverse straight leg raising test

This test is a way of eliciting root stretch in the evaluation of high lumbar radiculopathy. The patient lies prone and the knee is pulled into maximum flexion or the examiner pulls upward on the extended knee to passively extend the hip. In the bent knee pulling test, the patient’s knee is flexed, and the examiner pulls upward on the ankle while pushing the buttock forward. In all these variations, a normal individual should complain only of quadriceps tightness (Figure 8). With disc disease, there is a pain in the back or in the femoral nerve distribution on the side of the lesion (2).

##### Slump test

**Figure 9:**
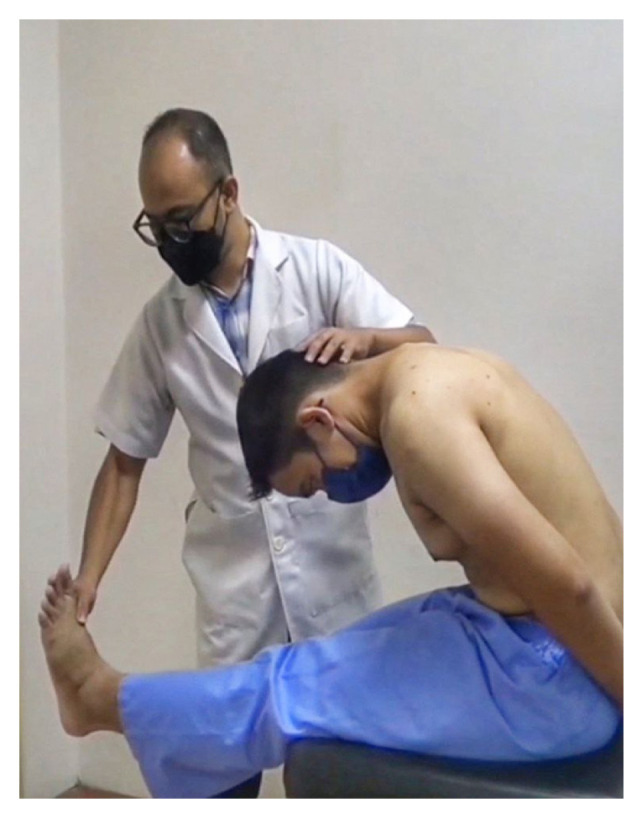
Slump test

Assess lumbar radiculopathy due to disc herniation in patients with low-back pain. The patient is seated with hands behind their back to achieve a neutral spine (Figure 9). The first step is to have the patient slump forward at the thoracic and lumbar spine. If this position does not cause pain, have the patient flex the neck by placing the chin on the chest and then extend one knee as much as possible.

If extending the knee causes pain, have the patient extend the neck into a neutral position. If the patient is still unable to extend the knee due to pain, the test is considered positive. If extending the knee does not cause pain, ask the patient to actively dorsiflex the ankle. If dorsiflexion causes pain, have the patient slightly flex the knee while still dorsiflexing. If the pain is reproduced, the test is considered positive (16).

#### Nerve Roots

##### Dermatome

**Figure 10:**
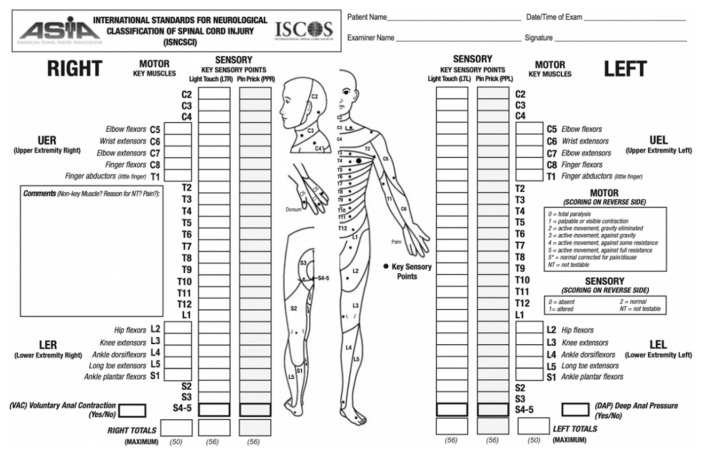
International Standards for Neurological Classification of SCI (ISNCSCI) Worksheet 2019. (Source: American Spinal Injury Association)

International Standards for Neurological Classification of Spinal Cord Injury (ISNCSCI) or commonly known as the ASIA Chart was developed by the American Spinal Injury Association (ASIA) as a universal classification tool for spinal cord injuries based on a standardised sensory and motor assessment (Figure 10).

Dermatome examination can be tested using light touch and pin-prick at the sensory points readily located in relation to bony anatomical landmarks at C2 to S5.

### Straight Leg Raising (Lasègue) Test Remains the Mainstay in Detecting Radicular Compression

The test is performed by slowly raising the symptomatic leg with the knee extended. During straight leg raising (SLR), tension is transmitted to the nerve roots between 30° and 70° and the pain increases. Pain at less than 30° raises the question of non-organicity and some discomfort and tightness beyond 70° are routine and insignificant. There are various degrees or levels of positivity. Ipsilateral leg tightness is the lowest level, pain in the back is more significant and radiating pain in the leg is highly significant (2).

### Deep Tendon Reflexes

Lastly, we finished our thoracic and lumbosacral examination by testing the reflexes (Table 4).

### Superficial Cutaneous Reflexes

#### Superficial Abdominal Reflexes

**Figure 11:**
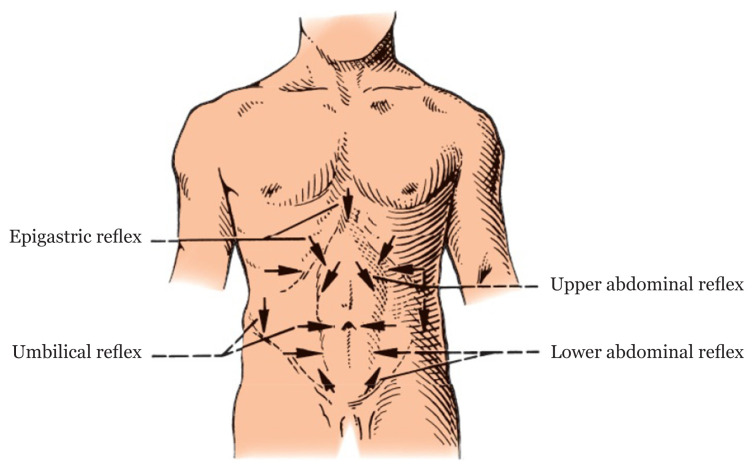
Superficial abdominal reflexes (2)

The response can be divided into upper abdominal and lower abdominal reflexes. The umbilicus is at the level of T10. The anterior abdominal wall can be divided into four quadrants by vertical and horizontal lines through the umbilicus (Figure 11). Light stroking or scratching in each quadrant elicits the response while pulling the umbilicus in the direction of the stimulus. The response is a quick, flicking contraction followed by immediate relaxation. The response is mediated in the upper quadrants (supraumbilical reflexes) by the intercostal nerves (T7–T10). In the lower quadrants (infraumbilical or suprapubic reflexes) by the intercostal, iliohypogastric and ilioinguinal nerves (T10 to upper lumbar segments) (2).

### The Superficial Reflexes of The Lower Extremities

#### The Cremasteric Reflex

This reflex is elicited by stroking or lightly scratching or pinching the skin on the upper, inner aspect of the thigh. The response consists of a contraction of the cremasteric muscle with a quick elevation of the homolateral testicle. The innervation is through the ilioinguinal and genitofemoral nerves (L1–L2) (2).

#### The Gluteal Reflex

A contraction of the gluteal muscles may follow with the stroking of the skin over the buttocks. The gluteus maximus is innervated by the inferior gluteal nerve (L4–S2) and the skin of this area is innervated by the cutaneous branches of the posterior rami of the lumbar and sacral nerves (2).

#### The Plantar Reflex

Stroking the plantar surface of the foot from the heel forward is normally followed by plantar flexion of the foot and toes. Flexion is the normal response after the first 12 months to 18 months of life. This reflex is innervated by the tibial nerve (L4–S2) (2).

#### The Superficial Anal Reflex

The cutaneous anal reflex (anal wink) consists of the contraction of the external sphincter in response to stroking or pricking the skin or mucous membrane in the perianal region. The reflex is mediated by the inferior haemorrhoidal nerve (S2–S5) (2).

#### Bulbocavernosus Reflex

The stimulus is delivered to the glans penis or clitoris (clitoroanal reflex). The response is best palpated with a gloved finger in the rectum. The bulbocavernosus reflex is primarily useful in assessing the integrity of the cauda equina, lower sacral roots and conus medullaris (2).

## Discussion

Thoracic and lumbosacral spine examinations started as the patient open the door and walk into the clinic room. From there, we can observe the patient’s gait or abnormality of posture. Then, we can proceed with palpation to check for any local tenderness. Range of movement can be divided into active and passive while a range of motion can be assessed by eyeballing. This, however, is a crude method of measurement and is fallible to the observer bias. Besides that, it is not an objective, accurate or reproducible measure of the spinal vertebrae motion.

The BROM unit consisted of two plastic frames. The frame used to measure lumbar spine sagittal plane motion consisted of an L-shaped slide arm that was free to move within a notch of the frame during sagittal plane measurements. The frame was secured to the subject by two Velcro straps. The second frame was designed to obtain measurements of both frontal and transverse plane motions. This frame has two measurement devices attached to it. One is a gravity goniometer with a freely moving pointer that moves about an axis at the centre of a protractor scale while the other is a compass. These two are arranged orthogonal to each other. During trunk rotation measurements, the unit requires a magnetic yoke mounted to the pelvis (4). It is a reliable method of measurement, far more than simple visual estimations.

A potential source of error with the BROM II device is slippage of the device over the sacrum during measurements of lumbar flexion and extension and angle of pelvic inclination. These measurements may be improved by securing the device firmly to the pelvis and not allowing the subjects or patients to wear clothing with lycra or silk textures (8). Since the flexion-extension unit of the BROM II device is secured to the subject’s pelvis using Velcro straps, the potential for movement error is greater than it is for the unit measuring lumbar rotation and lateral flexion.

Apart from that, we also discussed specific tests to identify spine pathology arising from the specific anatomy starting from the skin, muscles, ligaments, facets, discs, bone and nerves. This can guide the clinician to identify pathology from every patient, which in turn, will help the clinician to plan further investigation and imaging to treat the patient.

An example of a special test performed is the modified Schober’s test. It is used to determine if there is a decrease in the lumbar spine range of motion. The original Schober’s test is done by drawing a horizontal line at the centre of the lumbosacral junction followed by a second horizontal line marked 10 cm above the first line. Whereas in the modified Schober’s test, two marks are drawn 5 cm below and 10 cm above the first landmark to increase accuracy in measuring the range of motion. Although the modified Schober’s test reflected spinal mobility better than the original Schober’s test, both seemed to poorly reflect lumbar spine angular motion.

## Conclusion

A consistent and standardised method should be implemented when evaluating the range of motion of the thoracic and lumbosacral spine. This is to ensure that interobserver variability can be reduced and reliable monitoring of progress or deterioration of thoracic and lumbosacral range of motion can be provided. The specific special test can guide us to localised specific spine anatomy. To date, there are no comprehensive guidelines regarding the examination of the thoracic and lumbosacral spine and how to identify spine pathology based on the specific test. We hope that this examination technique can help the clinician treat their patient and help the student to learn the correct technique. We also prepared a video on thoracic and lumbosacral spine examinations for a better understanding of the examination technique.

A video (https://youtu.be/t3qW1g7WGm0) has been produced to demonstrate the examination techniques explained in this article.

## Figures and Tables

**Table 1:** Deep muscles of the back with functions and innervations (5)

Muscles	Functions	Innervations
Iliocostalis lumborum	Extension of the spine Lateral bending of the spine (muscles on one side)	L1–L5 spinal nerves, dorsal rami (variable)
Longissimus thoracis	Extension of the spine Lateral bending of the spine to the same side (muscles on one side)	Tl–Ll spinal nerves (dorsal rami)
Spinalis thoracis	Extension of the spine	T1–T12 (variable) dorsal rami
Semispinalis thoracis	Extension of the thoracic spine	T1–T12 spinal nerves (dorsal rami), variable
Multifidus	Extension of the spine Lateral bending of the spine (muscle on one side) Rotation to the opposite side	Spinal nerves (the whole length of the spine), segmentally (dorsal rami)
Rotatores thoracis	Extension of the thoracic spine Rotation to the opposite side	T1–T12 spinal nerves (dorsal rami)
Rotatores lumborum	Extension of the spine Rotation of the spine to the opposite side	L1–L5 spinal nerves (dorsal rami) (highly variable)
Interspinales thoracis	Extension of the spine	T1–T3; T11–T12 (irregular) spinal nerves (dorsal rami)
Interspinales lumborum	Extension of the spine	L1–L4 spinal nerves (dorsal rami), variable
Intertransversarii thoracis and lumborum	Extension of the spine (muscles on both sides) Lateral bending to the same side (muscles on one side) Rotation to the opposite side	T1–T12, L1–L5 spinal nerves (dorsal rami)
Quadratus lumborum	Extension of the lumbar spine (muscles on both sides) Lateral bending of the lumbar spine to the same side (pelvis fixed)	T12–L3 spinal nerves (ventral rami)

**Table 2:** Muscles of the abdomen with functions and innervations (5)

Muscles	Functions	Innervations
Obliquus externus abdominis	Flexion of the trunk (bilateral muscles) Rotation of the trunk to the opposite side (unilateral) Lateral bending of the trunk (unilateral) Tilt pelvis posteriorly Elevate pelvis (unilateral)	T7–T 12 spinal nerves (ventral rami)
Obliquus internus abdominis	Flexion of the spine (bilateral) Lateral bending of the spine (unilateral) Rotation of the trunk to the same side (unilateral) Elevation of pelvis	T7–T 2 spinal nerves (ventral rami) L1 spinal nerve (iliohypogastric and ilioinguinal branches) (ventral rami)
Rectus abdominis	Flexion of the spine (draw symphysis and sternum toward each other) Posterior tilt of the pelvis	T7–T12 spinal nerves (ventral rami)

**Table 3:** Myotome

Action	Muscle	Nerve Root	Nerve
Finger abduction	Interossei muscles	T1	Ulnar nerve
Thumb abduction	Abductor polices brevis	T1	Median nerve
Hip flexion	Psoas	L2	Femoral
Hip extension	Gluteus maximus	L5/S1	Inferior gluteal nerve
Knee flexion	Hamstrings	L5	Sciatic
Dorsiflexion	Tibialis anterior	L4/5	Deep peroneal
Plantar flexion	Gastrocnemius	S1/2	Tibial nerve

**Table 4:** Thoracic and lumbosacral examination by testing the reflexes

Reflex	Segmental level	Peripheral nerve
Quadriceps	L3–L4	Femoral
Achilles	S1	Sciatic
